# Mental health service accessibility, development and research priority setting in Cambodia - a post-conflict nation

**DOI:** 10.1186/s12913-023-09187-z

**Published:** 2023-02-22

**Authors:** Alan Maddock, Nil Ean, Anne Campbell, Gavin Davidson

**Affiliations:** 1 School of Social Policy, Social Work and Social Justice, University College Dublin, Dublin, Ireland; 2 School of Psychology, Royal University of Phnom Penh, Phnom Penh, Cambodia; 3 School of Social Sciences, Education and Social Work, Queen’s University Belfast, Belfast, Northern Ireland, UK

**Keywords:** Mental health, Service provision, Access, Health services, Policy, Research priorities

## Abstract

**Background:**

The limited health and social care infrastructure that existed in the 1970s in Cambodia was destroyed due to the Khmer Rouge. Mental Health service infrastructures have developed in Cambodia in the last twenty five years, however, they have been shaped significantly by very limited funding being made available for human resources, support services and research. The lack of research on Cambodia’s mental health systems and services is a significant barrier to the development of evidence-based mental health policies and practice. In order to address this barrier, effective research and development strategies are needed in Cambodia, which are based on locally well-informed research priorities. There are many possibilities for mental health research in LMIC countries such as Cambodia, therefore focused research priorities in these areas are needed to guide future research investment. This paper is the result of the development of international collaborative workshops, which focused on service mapping and research priority setting in the field of mental health in Cambodia.

**Methods:**

A nominal group technique was used to gather ideas and insights from a range of key mental health service stakeholders in Cambodia. Results: The key issues in service provisions for people with mental health issues and disorders, the interventions and programmes of support available, and currently needed, were identified. This paper also identifies five key mental health research priority areas which could form the basis for effective mental health research and development strategies in Cambodia.

**Conclusion:**

There is a clear need for the Cambodian government to devise a clear policy framework for health research. This framework could focus on the five research domains identified in this paper and could be incorporated within its National Health Strategic plans. The implementation of this approach would likely lead to the development of an evidence base which would allow the development of effective and sustainable strategies for mental health problem prevention and intervention. This would also contribute to promote the Cambodian government’s capacity to take the deliberate, concrete, and targeted steps necessary to address the complex mental health needs of its population.

## Introduction

### Mental health services structure in Cambodia

Cambodia is a lower middle-income country (LMIC) in South-East Asia with an estimated population of 15.6 million, of whom 42% are less than 15 years old [1]. From 1975 to 1979, Cambodia was ravaged by the Khmer Rouge, with as many as two million Cambodians dying from starvation, disease, or execution [2]. The limited health and social care infrastructure that did exist during this time was destroyed [3]. Mental Health service infrastructures have developed in the last twenty five years but these have been shaped by the very limited funding available for human resources and research [1, 2, 4]. The Cambodian government’s total health expenditure was 1.5% of GDP in 2013, lower than most LMICS in the region [5], with approximately 0.02 to 1% of the national health budget being allocated to mental health in 2011 [6, 7]. The number of psychiatrists in Cambodia is approximately 60 (1 per 260 000 people) [1] compared with 7068 (1 per 9300 people) in the UK [8]. The mental health services available are generally delivered in public and private hospitals in Phnom Penh, Cambodia’s capital city [9]. There are limited community mental health services that exist outside of Phnom Penh. Furthermore, mental health services are completely inaccessible to 68% of the population that live in rural areas [9, 10]. This often means that traditional healers are often the first point of contact that persons, or the family of persons with mental health issues and disorders encounter when trying to access mental health support [11, 12].

### Lack of mental health research

The Global Forum for Health Research [13] has long highlighted the major imbalance between the magnitude of mental health problems (especially in LMICs) and the resources devoted to addressing them. This is the 10/90 gap, that is, only 10% of global spending on health research is directed towards the problems that primarily affect the poorest 90% of the world’s population [9, 14]. Worldwide, a driving factor behind the recognition of mental health as a global public health concern has been the proliferation of epidemiological research documenting the considerable burden of mental health issues in all world regions [3, 6]. Though there is some published literature, describing how poor mental health is likely to have a significant impact on the social and economic growth of Cambodia [9, 15], there is a scarcity of mental health research in Cambodia [9]. There is very little research on factors that might influence the mental health of Cambodians, and it remains unclear which mental health interventions are the most effective in local contexts for mental health issues or major disorders [16]. This is particularly relevant, as a number of the international services that are available have been criticized for being culturally insensitive and not fully integrating with the local community, reducing their effectiveness [9]. This lack of research creates major barriers to successful mental health policy development, implementation and sustainable change in Cambodia [9].

### Research priorities

The gap between research and public health priorities has consistently been identified as a challenge for researchers and policymakers worldwide [17, 18]. This gap also exists in Cambodia, where the limited body of knowledge produced is driven by international funding priorities, rather than local public health needs [19]. Closing the gap between research, policymaking, and practice is likely to be central to enhancing the capacity of the Cambodian government to take the deliberate, concrete, and targeted steps necessary to address the complex needs of its population, through the development and implementation of evidence-based mental health policies [9, 16, 20–22]. This is more likely to be achieved through the development of effective research and development strategies, along with increased funding for human resources and research capacity building activities [21, 23–26].

Effective research and development strategies require well-informed research priorities based on evidence of need [27, 28]. There are many possibilities for mental health research in LMIC countries such as Cambodia, which are beyond the capability of any one government or agency to fund, therefore focused research priorities in these areas are needed to guide future research investment [21]. Publicly funded research and health-care systems should prioritise research questions that matter primarily to patients, their carers, health-care professionals and providers [29]. In order to ensure that such research agendas are aligned with priorities of these stakeholder groups, workshops and meetings between relevant professional groups and policymakers in Cambodia are necessary [16]. Patient, professional and policymaker involvement in research prioritization would help to close the gap between research, policymaking, and practice, as it would allow the relevant and quality research topics, which would help to inform best practice approaches in mental health in Cambodia, to be identified [30]. The early involvement of the government and professional and patient stakeholder groups might also encourage a sense of ownership of the research process, leading to greater implementation of research findings in policy and practice [31, 32]. The refocusing of research priorities in line with local patient and carer concerns is also likely to improve the quality of data collected, making the research process more efficient and impactful [31, 33–36].

## Methods

### Setting

This data contained in this paper came from the development of an international collaboration between a range of policymakers, governmental agencies, NGO partners and academics, from a range of discipline areas, who specialise in the areas of mental health and disability. This group had a particular interest in identifying the best way to meet the needs of people with mental health issues, and a co-morbidity of mental health and disability issues in Cambodia. The group included members of the disciplines of Social Work in University College Dublin (then employed at Queen’s University Belfast (QUB)), Social Work, Psychology, Social Policy and Psychiatry at QUB, the Department of Rehabilitation in Jonkoping University, Sweden, the Royal University of Phnom Penh, the Department of Disabilities and Social Affairs in Cambodia, the Department of Mental Health and Substance Abuse within the Ministry of Health in Cambodia and the NGO sectors in both mental health and disability in Cambodia. This group met over 5 days as part of a service mapping and research priority setting workshop in order to: 1) identify the key issues which may limit the extent to which people with mental health issues and disabilities receive appropriate support, 2) identify the services and interventions currently available, 3) identify the key services and interventions that need to be implemented, and 4) to develop a list of the most important research priorities in mental health, which would inform the development of effective and sustainable strategies for prevention and intervention in Cambodia.

### Technical working groups and context

In order to meet the aim of this service mapping and research priority setting process, we utilized the mapping health service provision steps model outlined in Price et al. [34]. The first two steps of this model included: 1) defining which target services we wanted to map (which were the mental health services in Cambodia), and 2) we invited the most relevant and informed expert Cambodian professional and patient advocacy stakeholders and policymakers to both workshops. Ideally, there would have been a clear definition of what constitutes expertise and a sampling strategy for locating experts who meet it. However, sometimes experts, as in this case, are harder to define and there was no clear sampling frame to follow in this context [35]. In order to identify and invite the participants to both workshops, AM used purposive and snowball sampling.

### Recruitment process

Prior to sending out invitations, AM conducted a review of the literature on mental health and disabilities in Cambodia. This allowed AM to identify research active Cambodian academics, clinicians, policymakers, and government officials. AM contacted these stakeholders and asked them: (a) if they would be interested in being a part of the workshop, and (b) to identify any other participants that AM should contact. AM also identified—with support from a NGO supporting persons with physical impairments (Exceed Worldwide) in Cambodia, which is headquartered in Northern Ireland—other key stakeholders that AM should invite. As part of this process, AM contacted NE, who is a Lecturer in Psychology in RUPP and the clinical psychological lead of a mental health NGO in Cambodia, which has formal links to the Department of Mental Health in Cambodia. NE also has many years’ experience working within the disability sector in Cambodia. NE verified AM’s list of participants to invite and then identified and invited other relevant stakeholders to the workshop on behalf of AM. Formal invitations to attend the workshop were sent out to the identified participants along with an information pack via email. The participants that did not respond to the email invitations initially were contacted subsequently by phone. The information pack was developed for participants to gain a greater understanding of the reason for hosting the event, the structure and purpose of each day, and the mental health themes which would be under discussion. The information pack also outlined that the two workshops would be collaborative in nature, with the Cambodian contributors viewed as experts, setting the agenda where possible [36]. Forty-four participants (24 males and 20 females) who were either Cambodian (*N* = 42) or worked in a Cambodian NGO (*N* = 2) participated. The participants consisted of 2 government officials (1 junior government minister and 1 deputy to a government minister), 10 disability professionals (comprising of 4 social workers, 2 prosthetists, 2 social care workers, and 2 nonprofessional workers), 2 disability advocacy professionals from 2 different organisations, 23 mental health professionals (15 social workers, 2 adult psychiatrists, 3 psychologists, 2 occupational therapists, 1 child and adolescent psychiatrist), 4 mental health academics (2 psychology, 2 social work), 3 disability academics (3 social work) [36]. To the author’s knowledge, there were no mental health patient advocacy groups available in Cambodia that could be invited, to attain the service user perspective. There were eight participants from the Queen’s University Belfast delegation (including the lead author who now works for University College Dublin), who were academics in social work (6), psychology (1), psychiatry (1) with another academic in disability rehabilitation from Jonkoping University in Sweden. The role of this group of academics was to learn from their Cambodian colleagues about what services were available and what the gaps in provision were. The hope being that the collective group collaboration could then identify, what the most important research priorities and questions in mental health in Cambodia were, which the collaborative group could then seek funding to answer.

### Data collection and analysis

The author’s then adapted step 3, 4 and 5 of the Price et al. [34] i.e., design data collection, data collection and data analysis and handling, to fit the fit the workshop format. In order to collaboratively identify the services that were accessible to people with mental health issues along with the most important research priorities in each context in Cambodia, a nominal group technique (NGT) was used. The nominal group process is a structured meeting, which seeks to provide an orderly procedure for obtaining qualitative information from expert groups who are most closely associated with a problem area [37]. This technique was selected as it helps to prevent dominance of views by individual participants or of particular perspectives, and to encourage quieter members to participate. This approach was felt to be particularly relevant in a Cambodian context where the cultural norms can lead to a strict adherence to hierarchy in relationships surrounding age, gender, and professional title [38].

### Individual questionnaire

On each of the 5 days, several government officials and NGOs provided presentations about the nature of their work and the needs of the people they worked with. Several academics also provided presentations on the nature of their work in the areas of mental health and disability. Each of the workshop’s participants were asked to fill in a questionnaire, devised by AM, NE, AC and GD, which contained the following questions: 1) What are the key issues relevant to mental health in Cambodia?, 2) What services and interventions are currently available for people with mental health issues in Cambodia?, 3) What other interventions are needed in mental health?, (4) What are the priorities for research about mental health in Cambodia?, (5) What are the priorities for research about the interaction of disability and mental health in Cambodia?, (6) What are the barriers for your organisation being involved in research?, and (7) What would help support your organisation to be involved in research? The data generated from these questionnaires were analysed using directed content analysis [39]. This process involved the authors reviewing the qualitative data, before applying a number of overarching research topic categories which this data could fit within [39].

### Stakeholder discussion

After completing the individual questionnaire exercise, participants were then divided evenly into groups reflecting their field of expertise e.g., disability or mental health, or those who worked with people with co-occurring issues. The participants engaged in discussions in their relevant groups and generated research ideas as a form of mind mapping/collaborative activity. After completing this task, group facilitators (AC and GD) brought the groups together. Each group was given the chance to voice their ideas, which was then recorded by a scribe (AM). These ideas were distilled as the discussions progressed and refined into research topics or specific ideas to be taken forward. All experts presented their views on research needs, and discussion followed until consensus was reached. The material derived from these discussions were collapsed into a main document [40]. Taking direction from the participants, the overall task facilitators (AC and GD) transferred subsequent research questions and ideas for studies under these research domains onto a flipchart. Twenty five research priorities were identified, which fit under the following five research priority themes [41]: 1) Problem – estimating prevalence and burden, 2) Cause – with a focus on conceptual understandings of mental health in the Cambodian context, 3) Solution—effectiveness of interventions (new and existing), 4) Implementation – focusing on community need gaps in service planning, 5) Social Inclusion/Exclusion – exploration of individual and group experiences. The full list of research topics are contained in Table 1, in the results section below.

### Follow up survey

To further ensure that all participants had an opportunity to contribute in an equal manner and to identify the most important research priority categories, outlined in Table 1 below, the participants who provided contact details were invited by email by AM to complete an online survey using an embedded survey link. Participants were asked to select which of the topics, within each of the categories agreed on in the workshop e.g., problem, prevalence, were most important in their view, ranking each in order of preference.

Step 6 of Price et al. [34], communicating of findings, will follow in the results and discussion section, with step 7 hosting the service maps, occurring through the publication of this paper.

## Results

### Mental Health Services

The participants who completed the individual questionnaires (*N* = 44) consistently identified similar issues, which limit the extent to which people with mental health issues receive appropriate support. These were: 1) a chronic lack of access to mental health services, particularly in rural areas due to the fact that health services are hospital-based and highly centralized in a few major cities, 2) a shortage of trained personnel within the limited services that do exist, particularly those who could offer psychosocial supports, 3) poor mental health literacy amongst the local population and awareness of available services, 4) people with mental health issues experiencing very high levels of stigma and discrimination, 5) limited co-ordination and collaboration amongst professions and service providers, 6) limited policy and clinical guidelines, 7) little or no screening and/or early intervention services, 8) traditional healers being the front line contact for people with mental health issues.

### Services and interventions currently available

The participants were able to identify a wider range of services available to meet the needs of the local community in Phnom Penh. These were: 1) The Khmer-Soviet Friendship Hospital, 2) Transcultural Psychosocial Organisation (NGO working with adults), 3) The Center for Child and Adolescent Mental Health (CCAMH). The participants identified that there are some referral hospitals in the provinces which offer mental health supports. These participants advised that most of the mental health supports that are available are provided by under resourced NGOs, who meet the mental health needs of the local population as best they can e.g., using basic counselling with person-centred techniques.

### Services and interventions needed

The participants identified a range of similar services and interventions that were needed to improve the quality and content of mental health services available to Cambodian people. These were: 1) decentralized, accessible and affordable mental health services at primary, secondary and tertiary care levels, 2) increased training of and access to other mental health professionals (e.g., psychologists, social workers, counsellors) as part of the mental health system, 3) increased self-care and stress coping interventions for professionals and carers, 4) increased access to other non-pharmacological therapies which are culturally informed e.g., psychoeducation, mindfulness-based interventions, cognitive behavioural therapy (CBT), family counselling/support, art, play, and music therapy, 5) group mental health interventions which can delivered at a low cost by non-specialists, 6) public mental health literacy campaigns – particularly for students in mainstream education, and 7) older age services.

The data from the individual questionnaires generated 17 different research priority themes in the areas of mental health, and mental health and disability.

### Mental health research priorities

The participants in both workshops identified a number of key mental health research priorities which were: 1) prevalence studies of mental health issues (anxiety, depression and schizophrenia) in the community, school and workplace to provide clear directions for service planning, 2) the effectiveness of interventions for: mental health treatment and recovery, prevention and promotion, communitybased mental health care (e.g., task shifted mental health interventions delivered by other professionals), 3) studies on help-seeking behaviour which might help develop a stepped-up care model and referral pathways, 4) randomised controlled trials (RCT) of self-care protocols for mental health service staff e.g., mindfulness for self-care, 5) mental health tools validation for assessment and research (clinical and field use e.g., mental health screening), 6) RCTs of WHO protocols e.g., group interpersonal therapy, 7) health system research with the objective of integrating mental health services within the wider health system, 8) studies on the social determinants (e.g., poverty) of mental health problems, 9) research on the impact of mental health problems among particularly at risk groups such as victims of trafficking, and left behind children, 10) action-research on the use of psychotropic drugs at health centre level, 11) research on the complex comorbidities of mental health and addiction issues, 12) studies on the factors that have led to the high suicide rates among young people in Cambodia, 13) research on what the best clinical guidelines for mental health services are in Cambodia.

### Mental health and disability research priorities

The workshop participants identified several key research priorities in mental health and disability. These were: 1) prevalence studies on the levels of mental health issues in populations with disabilities (focusing on psychological distress), 2) effectiveness studies on interventions that will improve mental health, quality, and well-being of people with disabilities and/or their carers, 3) studies which explore the impact of a person’s disability on their own and their caregiver’s mental health, 4) studies which explore the coping skills of people with disabilities.

### Research barriers and facilitators

The workshop participants identified several barriers to research in Cambodia. The lack of research funding, of trained and motivated personnel, and clinical staff time available to conduct research were consistently cited as the biggest barriers to research. Other issues included: significant problems with both leadership and co-ordination across health care departments of the Cambodian government. In addition, the importance of research in some university programmes has not yet been acknowledged. There are also barriers to gaining ethics approval from the National Ethical Committee for Health Research e.g., the $400 application review fee (which is waived for applications from students). There is also a lack of questionnaires developed in Khmer and validated, or validated questionnaires translated and validated within the Cambodian context. An increase in research funding, coupled with training for key staff in research methods, along with increased collaboration with international universities were identified as key facilitators for increased research in Cambodia.

### Focused research priorities

To identify the most important research priorities, in each category area, participants were asked to select which of the topics, within each of the categories agreed on in the workshop e.g., problem, prevalence, outlined in Table 1 below, were most important in their view, ranking each in order of preference. To identify the top three research priorities in each category, any research topic that received a first preference vote received 20 points, a second preference vote received 10 points and a third preference received 5 points. The participants were also asked to rank from 1–5 which of the research categories should be focused on. Total points per research priority were calculated based on all responses (*N* = 17) and then research priorities were re‐ordered from highest to lowest. This scoring criterion has been used in similar published papers on research priority setting partnerships (e.g., Finer et al., 2018). The results for each category are presented in Tables 2, 3, 4, 5, 6 and an overall evaluation of which of the main priority areas were deemed to be the most important is presented in Table 7 below.

**Table 1:** Research topics identified

*Research categories*	*Research topics*
Problem – prevalence	• Mental health (MH) issues in children and young people
	• MH issues in adults
	• MH issues in children and young people with disabilities
	• MH issues in people with disabilities in adults
	• Drug and alcohol issues
Cause—conceptual understanding	• How MH issues are experienced
	• Understanding health seeking behaviours
	• Cultural conceptualisation of mental health symptoms and mental health disorders
Solution – effectiveness	• Community based psychological (e.g., cognitive behavioural therapy), social (e.g., poverty reduction supports) and psychosocial interventions (e.g., psychoeducation, peer support) and medical mental health care interventions (e.g., medication)
	• Self-care interventions for professionals
	• Mindfulness interventions for a range of mental health issues and disorders (e.g., depression)
	• Early childhood interventions
	• Grants/loans in creating employment/educational opportunities
	• Cognitive behavioural therapy
	• Support programmes for substance misuse (e.g., relapse prevention)
Implementation – gaps	• Pathways to engagement in services for children and families
	• Identifying the best treatment pathways for mental health services with individuals with disabilities
	• Services for children who are “left behind” or orphaned and their carers
	• Exploring family experiences of caring for children with disabilities
Social inclusion/exclusion	• MH and disability – experience of abuse, stigma and discrimination
	• Exploring the need for the development of Mental Health legislation
	• Exploring and understanding the use of force in MH intervention
	• Stigma faced by parents of children who have MH problems
	• Rights of people who have MH and/or disability issue
	• Service access needs of people in rural areas

**Table 2:** Problem – prevalence results

*Problem-prevalence*	MH issues in children and young people	MH issues in adults	MH issues in children and young people with disabilities	MH issues in people with disabilities in adults	Level of drug and alcohol issues
1st Preference	8	8	4	3	3
2nd Preference	5	3	9	7	5
3rd Preference	2	2	2	4	2
Total	220	200	180	150	120

**Table 3:** Solution – effectiveness results

*Solution – effectiveness*	Community based psychological, sociological and psychosocial interventions and medical mental health care interventions	Self-care interventions for professionals	Mindfulness interventions	Early childhood interventions	Grants/loans in creating employment/educational opportunities	Cognitive behavioural therapy (CBT)	Support programmes for substance misuse
1st Preference	7	7	6	4	5	3	3
2nd Preference	5	4	4	7	4	8	6
3rd Preference	2	2	2	2	4	1	1
Total	200	190	170	160	160	145	125

**Table 4:** Cause—conceptual understanding results

*Cause—conceptual understanding*	How MH issues are experienced	Understanding health seeking behaviours	Cultural conceptualisation of symptoms and disorders
1st Preference	10	9	3
2nd Preference	5	7	9
3rd Preference	2	1	5
Total	260	255	175

**Table 5:** Implementation – gaps results

Implementation – gaps	Improve pathways to engagement in mental health services for children and families	Improve pathways to mental health services for individuals with disabilities	Implement support services for children who are “left behind” or orphaned and their carers	Implement services for people who are caring for children with disabilities
1st Preference	9	7	7	3
2nd Preference	5	8	6	8
3rd Preference	2	2	2	4
Total	240	230	210	160

**Table 6:** Social inclusion/exclusion results

Social Inclusion	MH and disability – experience of abuse, oppression	Development of Mental Health legislation	The use of force in MH interventions	Stigma faced by parents of children who have MH problems	Rights of people who have MH and/or disability issues	Service access needs of people in rural areas
1st Preference	5	7	5	3	1	2
2nd Preference	9	3	6	9	10	4
3rd Preference	2	2	3	1	2	6
Total	200	180	175	155	130	110

**Table 7:** Overall priority

Overall	Implementation	Solution	Social inclusion/exclusion	Problem	Cause
1st Preference	12	10	4	4	1
2nd Preference	3	3	7	6	8
3rd Preference	1	3	5	3	3
Total	275	245	175	155	115

## Discussion

### Mental health services

This paper identifies the key issues which may limit the extent to which people with mental health issues and disorders in Cambodia receive appropriate support, the services, and interventions currently available, the key services and evidence-based interventions that are currently absent from mental health services in Cambodia, and the most important mental health research priorities. In line with the findings of Jegannathan et al. [42] and Olofsson et al. [1], the key theme that emerged from the data from the individual questionnaires was one of limitation. The service infrastructures in mental health in Cambodia are characterised by chronic shortages of trained personnel to appropriately meet the needs of service user groups, and limited access to appropriate specialist services, for example, early intervention and gerontology services. The gaps identified are like those identified in other LMICs [43]. While trained professionals are essential to provide specialised services, one strategy to address the human resources shortage could be to provide tailored training to professionals from other sectors and allow them to administer basic interventions [44]. This practice is called task shifting and has been advocated for in the Cambodia context by Chhim [3]. A recent commentary on task shifting for managing gaps in mental health services indicated that it could facilitate early recognition of mental health issues, community engagement, enhanced access and uptake, stigma reduction and better adherence [45]. Similarly, the WHO recognizes task shifting as a viable strategy for increasing access to mental health services to underserved populations [46]. The participants also called for the limited services that are available to be decentralised and made more accessible to people who live in rural communities, agreeing with Seponski et al. [9]. The need for greater professional co-ordination and collaboration and cross disciplinary team working amongst the available professionals in mental health services, was identified as a key area in which improvements were needed, supporting Coton et al. [16]. Mental health issues e.g., depression, and disabilities often co-exist, therefore greater collaboration and integration of services would likely lead to early identification and treatment of mental health problems [46]. The incorporation of traditional healers and monks, who are often the first point of contact for persons with mental health issues and disorders in Cambodia, as part of such interprofessional collaborations would likely further support the early identification of mental health issues and disorders, while also supporting referral pathways for persons experiencing these problems [47].

### Research priorities and policy

A lack of evidence-based policies and clinical guidelines were also viewed as key limitations in mental health services. Clinical guidelines and evidence-based policies are statements with recommendations for practice rigorously developed based on systematic reviews of evidence and an evaluation of the benefits and harms of approaches [48]. The implementation of clinical guidelines and evidence-informed policy which is underpinned by evidence from high quality research projects conducted in Cambodia is likely to be key to the improvement of quality and consistency of care provided to people with mental health issues and disorders in Cambodia. The research priorities identified in this paper may provide important starting points from which to develop such projects.

### Research gaps

The participants in the workshop identified several research priorities, which are in line with a number of key research gaps which have already been identified in the literature. The identified need for more research to be conducted on the needs of children and adults with mental health and/or disability issues supports the works of a number of authors [17, 41, 49, 50]. The need for research examining the impact of living with a mental health and/or disability issue would have in the Cambodian context, again is well supported in the literature [51–54]. The workshop participants also identified that people with mental health issues and disorders suffer from high levels of stigma and discrimination due to their issue or impairment [6]. The workshop participants proposed that the reduction of stigma and discrimination could be addressed via government funding for mental health public literacy campaigns and inclusive education services for people with mental health problems. This approach would likely improve the lived experience of people living with mental health issues and disorders, by reducing the levels of stigma and discrimination experienced on a day-to-day basis and enhancing the quality of care available to both groups. This is due to the fact that stigma and discrimination have been associated with: decreased treatment effects, a higher probability of relapse [54], professionals being less willing to specialise in mental health [55], being a barrier to quality care and integration of mental health services within mainstream services [56].

### Research priority options

Mental health is clearly a low priority for government spending and as such the government’s need for research in this area has suffered as a result [9, 57]. The results of the prioritization exercise described in this paper, identified the top five research options related to implementation, solution, social inclusion/exclusion, problems, and causes [49]. The participants who completed the online surveys identified the need to focus research on exploring the feasibility of implementation and scaling-up of a range of potentially effective interventions for adults, young people, and carers of people with mental health issues and disabilities. This finding is in line with Coton et al. [16] and Hinton et al. [58], which both identified a clear lack of such research being conducted in these areas. Goyet et al. [19] outlined that from 2000–2012, there were only 53 health systems research publications. Maddock et al. [57] in a systematic review of psychological and social interventions for mental health issues and disorders in Southeast Asia (including Cambodia), found only two RCTs which were conducted in Cambodia. The participants also identified the need for large scale prevalence studies of the type and nature of impairments experienced by adults, children and young people with disabilities and mental health issues in Cambodia. The participants also identified the need within these studies to ascertain the type and extent of complex co-morbidities in both populations e.g., levels of alcohol and/or substance use. This supports findings from the previous work by Maramis et al. [17]. A prevalence study was conducted by the Department of Psychology at the Royal University of Phnom Penh (N = 2690) [7]. Perhaps unsurprisingly given the high rates of poverty and the historical context, mental health issues and disorders were found to very high e.g., Schunert et al. [7] found that 31.7% of female respondents were deemed to have a probable anxiety disorder. Despite being the first large scale research project of its kind in Cambodia, there were major limitations in this study, for example, an over presentation of female participants due to the lack of male participants during the harvest season and no clinical examinations took place during the data collection, meaning that the prevalence of mental disorders only remained probable. Due to the limited research being conducted in this area, there remains a need for large scale mental health prevalence studies in Cambodia. The development and implementation of data systems within government and large-scale NGO services could also help to capture the prevalence and nature of mental health issues and disorders in Cambodia in a consistent manner [17].

The participants in the workshop identified the need for research, which would increase knowledge on how mental health problems are experienced in Cambodia. The participants identified a clear need to understand more clearly how mental health is conceptualised by Cambodians and their health seeking behaviours. The participants identified that this knowledge could allow several evidence-based Westernised mental health interventions e.g., mindfulness-based interventions, and/or CBT, to be culturally adapted to the Cambodian context. The importance of this cultural adaptation to the local context was emphasised as being a potentially key facilitator of greater intervention effectiveness and of integration of Western services within local communities [9]. Our results highlight that though each of the five research domains identified in the priority setting exercise i.e., implementation, solution, social inclusion/exclusion, problems, and causes [41] – are clearly very important in their own right, each are also interconnected and there is a clear need for research across all five domains. In an environment with limited resources, where every investment decision must be strategic, with a view to establishing integrated systems of care and support, whilst simultaneously having the greatest impact in the short term, based on our findings, it might be wise initially to focus on research which improves the implementation of the limited services that are available to meet the needs of a wider cohort of persons with mental health and/or disability issues. This research could include how to best close the gap between service delivery and service access in Cambodia. Our findings suggest, that the next strategic focus should then be on evaluating the effectiveness of the interventions and programmes of support that are delivered within mental health services in Cambodia. There has been some small-scale research which has supported the potential effectiveness of Westernised interventions adapted to the Cambodian context. Hinton et al. [58] conducted a repeated measures study (N = 40) on the effect of a culturally adapted CBT intervention for treatment resistant PTSD and panic attacks, which incorporated mindfulness breathing exercises with Cambodian refugees versus delayed treatment in a cross-over design. Hinton et al. [58] found that this intervention reduced PTSD, panic attack severity, psychological distress, and generalized anxiety disorder status. This study’s findings are promising, however larger scale RCT studies, which replicate these findings, and test the effectiveness of other culturally appropriate interventions on other mental health disorders e.g., anxiety and depression, are clearly needed. It is clear that research topics which 1) examine the prevalence of mental health issues and disorders in Cambodia, 2) explore the social exclusion experienced by persons with mental health and disabilities, and 3) allow deeper insights into conceptual understanding of mental health, and health seeking behaviours are key strategic research priorities, but for the moment, based on our research findings, these could perhaps be focussed on at a later date.

### Protecting the limited staff that are available

The participants also identified the need for self-care interventions. The respondents outlined how mindfulness-based interventions could be adapted as self-care interventions for professional staff and carers. The lack of trained human resources available in mental health, can lead to the professionals that are available carrying exceptionally high caseloads [6]. If appropriate self-care strategies are not put in place by professionals, burnout is likely [59]. Professional burnout can lead to reductions in the human resources that are available, intervention effectiveness, and increases in unethical practices [59]. The implementation of evidenced-based culturally appropriate mindfulness self-care interventions, which have their roots within Buddhist doctrine, the formal religion in Cambodia [60], may help to ameliorate these issues. Integrating mindfulness interventions within the self-care of mental health professional groups (e.g., counsellors), has been shown to reduce burnout and improve practitioner well-being in Western contexts [61].

### Importance of learning from local stakeholders

The organization of the workshop and the identification of the key stakeholders would likely have been impossible without working with and learning from Cambodian professionals and organizations who had established networks in the fields of mental health and disability. The study procedure was both transparent and inclusive, ensuring involvement of a broad range of Cambodian researchers, policymakers, and practitioners. The need for such an approach was identified by [36], who identified the need for increased inclusive collaboration amongst key stakeholders in research, policy, and practice in Cambodia. The experts chosen for the workshops were highly targeted and to the author’s knowledge, the largest group to conduct such a research priority exercise to date in Cambodia. The use of a collaborative nominal group technique allowed the generation of many research priorities, so much so that a voting system had to be put in place, so that the highest priority goals for research could be set [36].

### Limitations

This paper discusses a qualitative inclusive approach to research that attempted to ensure that all voices are heard. This can be difficult in a research environment and it is inevitable that some voices might be lost. The facilitation of groups is an important feature of the nominal group technique, which requires patience and experience. All facilitators were experienced in group facilitation and management, and followed the objective mapping health service provision steps model outlined in [34], so that every effort was made to capture all services available and research ideas; the generation of so many research ideas support this view. The anonymous research priorities survey also provided the opportunity for people to anonymously choose the priorities for research, without the interference of policy makers or government officials, who might have their own agendas for research. The ranked data attained in this data collection process is limited in what it can tell us though, as differences in the order of priorities does not tell us anything about the degree of professional preference for each ranking [62]. We feel that the limitations of this research were kept to a minimum but acknowledge that some people may not have been able to participate as much as they would have liked. The research priorities that were suggested could be subject to expert opinion bias and may not reflect the research priorities of others who weren't involved. The way research priorities are identified however, particularly for research funding calls, also suffer from similar expert opinion biases. The experts in attendance did represent a significant proportion of the available professionals (and advocates for people with disabilities) in their respective fields in Cambodia (e.g., the only child and adolescent psychiatry in Cambodia was present, as were 2 out of the estimated 60 qualified psychiatrists in the country). It is thus unlikely that a different group of experts would have arrived at significantly different results.

### Conclusion

The development and implementation of tangible research projects, which focus on the outlined research priorities, through increased governmental and international research funding could help to close the current wide gap between mental health research, policy making and practice in Cambodia [5, 20]. For example, the development of screening questionnaires which are developed in Khmer and then validated would increase the validity and reliability of these measurement tools. The increased use of RCTs of locally developed mental health interventions, such as testimony therapy [63], could lead to more evidence-based practice and greater take up of mental health programmes due to their cultural sensitivity and relevance. There is a clear need for the Cambodian government to develop effective research and development strategies, and to devise a clear policy framework for health research, something which is currently absent [16, 21, 23, 24]. This framework could focus on the five research domains identified in this paper and could be incorporated within its National Health Strategic plans [18]. The implementation of this policy e.g., through financing activities which would enhance the research capacities of local academics and motivated professional personnel, would likely lead to the development of an evidence base which would allow the development of effective and sustainable strategies for prevention and intervention in mental health [5, 21]. This would also contribute to promote the Cambodian government’s capacity to take the deliberate, concrete, and targeted steps necessary to address the complex mental health needs of its population [5, 21].

## Data Availability

If you would like to request the data from this study, please contact the corresponding author.
