# Correction: Mental health service accessibility, development and research priority setting in Cambodia - a post-conflict nation

**DOI:** 10.1186/s12913-023-09253-6

**Published:** 2023-03-08

**Authors:** Alan Maddock, Nil Ean, Anne Campbell, Gavin Davidson

**Affiliations:** 1 School of Social Policy, Social Work and Social Justice, University College Dublin, Dublin, Ireland; 2 School of Psychology, Royal University of Phnom Penh, Phnom Penh, Cambodia

**Correction to**: *BMC Health Services Research* (2023) 23:183. 10.1186/s12913-023-09187-z.

Following publication of the original article [1], an error was identified in the corresponding authorship assignment due to a typesetting mistake: corresponding authorship was assigned to Anne Campbell instead of to Gavin Davidson.

The author group has been updated above and the original article [1] has been corrected. The publisher apologises to the authors and readers for the inconvenience caused by the error.
